# Diastasis recti in the Beninese population: Cross-sectional study from normal values to diagnosis

**DOI:** 10.4102/sajp.v78i1.1776

**Published:** 2022-11-15

**Authors:** Yollande S. Djivoh, Toussaint Kpadonou, Thierry Puttemans, Dominique De Jaeger

**Affiliations:** 1 Institute of NeuroSciences (IoNS), Laboratory of Biomechanics and Physiology of Locomotion, Faculty of Motor Sciences, Catholic University of Louvain, Louvain-la-neuve, Belgium; 2 University Clinic of Physical Medicine and Rehabilitation of CNHU-HKM, Faculty of Health Sciences, National University of Abomey-Calavi, Cotonou, Benin; 3 Department of Radiology, St-Pierre Clinic, Ottignies, Belgium

**Keywords:** interrecti distance, African population, abdominal muscles, diastasis recti, linea alba

## Abstract

**Background:**

Diastasis recti is diagnosed when the interrecti distance (IRD) is larger than a threshold value. Published thresholds were measured at rest with ultrasound while in Benin physiotherapists use calipers during abdominal contraction.

**Objectives:**

The aim was to define IRD threshold values measured with calipers in Beninese participants in order to diagnose diastasis recti in a clinical environment and identify women needing abdominal rehabilitation.

**Method:**

Interrecti distance was measured using ultrasound and calipers. Linea alba stiffness was assessed by palpation, abdominal strength and endurance by manual testing. In men and nulliparous women, IRD threshold values were defined as IRD P90. In postpartum women, IRD P80 and a threshold defined with a receiver operating characteristics (ROC) curve based on linea alba stiffness were used. In these women, abdominal strength and endurance were compared depending on IRD threshold and linea alba stiffness with a Mann Whitney test.

**Results:**

In 391 Beninese participants, the IRD threshold measured with calipers was 17 mm in men, 15 mm in nulliparous and 18 mm (15 mm with ROC curve) in postpartum women. Postpartum women with an IRD above 18 mm had significantly lower abdominal strength. Those with a slack linea alba had significantly lower abdominal strength and endurance.

**Conclusion:**

The defined IRD threshold values can be used in a Beninese clinical environment. Future studies should confirm whether they can be applied to other African populations.

**Clinical implications:**

Abdominal rehabilitation should be recommended to postpartum women whose IRD is above the threshold values but also in cases of slack linea alba and poor abdominal function.

## Introduction

During pregnancy, there is a high risk of developing diastasis recti (Gilleard & Brown 1996; Sperstad et al. 2016) defined as an increased separation of the two rectus abdominis muscles along the linea alba (Akram & Matzen 2014; Gluppe, Engh & Bø 2020). The linea alba is a fibrous structure formed by the fusion of the aponeuroses of the anterior abdominal wall muscles (Axer, Keyserlingk & Prescher 2001). It stretches from the xiphoid process to the symphysis pubis (Boissonnault & Blaschak 1988). According to Gilleard and Brown (1996), in pregnant women, hormonal changes and increased uterine volume decrease the linea alba stiffness and increase its width measured as the interrecti distance (IRD).

Normal values of the IRD have been determined in nulliparous and primiparous women and threshold values have been proposed, above which the diastasis recti is diagnosed (Beer et al. 2009; Mota et al. 2018; Qu et al. 2021). For postpartum women, the 80th percentile (P80) of the IRD measured in 84 primiparous women at 6 months postpartum was used by Mota et al. (2018) as the threshold value for the diastasis recti. They reported a P80 value of 24 mm at 5 cm above the umbilicus and of 28 mm at 2 cm above the umbilicus. The 90th percentile (P90) of IRD values has been proposed for nulliparous women to define this threshold. At 3 cm above the umbilicus, the P90 value of the IRD is 22 mm according to Beer et al. (2009) and 14 mm according to Qu et al. (2021). In these two studies, nulliparous women were very similar regarding the sample size (150 vs. 116), participants’ average age (29 ± 6 vs. 30 ± 5 years old) and body mass index (BMI) (22 ± 4 kg/m^2^ vs. 21 ± 3 kg/m^2^). However, one study was conducted in Western Europe (Beer et al. 2009) and the other in China (Qu et al. 2021). This suggests that a threshold value measured in one population might not be valid for another one. Studies from Africa on diastasis recti in postpartum women do not report on IRD threshold values. These studies focused on the prevalence of the diastasis recti, the resolution of the diastasis recti and the correlation between IRD and parity, waist and hip circumference (Alamer, Kahsay & Ravichandran 2019; Igwe & Okoye 2020; Igwe, Okoye & Chukwu 2020; Ojukwu et al. 2021).

In the literature, IRD threshold values are all measured at rest in the supine position using ultrasound (Beer et al. 2009; Mota et al. 2018; Qu et al. 2021). In the Beninese clinical environment, palpation methods like finger width or measurements with calipers are more commonly used than ultrasound. Palpation methods require a slight contraction of the rectus abdominis muscle and are therefore performed in a ‘head lift position’ or during curl-up, not at rest. Because rectus abdominis contraction reduces the IRD (Chiarello, McAuley & Hartigan 2016; Gluppe et al. 2020; Mota et al. 2015; Pascoal et al. 2014; Sancho et al. 2015), smaller IRD values are expected in a head lift position or during curl-up than at rest (Gluppe et al. 2020). So far, the IRD threshold values specifically measured in a head lift position or during curl-up are not known. The first two aims of our study were to describe IRD values for men, nulliparous and postpartum women in the Benin Republic, and to define IRD threshold values measured at rest with ultrasound and in a head lift position with calipers. The IRD threshold values measured at rest with ultrasound will be compared with those already published in Caucasian and Chinese samples. Those measured in a head lift position with calipers will be proposed for use in the clinical environment.

A strong correlation was observed between the IRD width and the abdominal muscle strength in patients with diastasis recti (Gunnarsson et al. 2015), and lower abdominal muscle strength and endurance were observed in postpartum women with diastasis recti compared to those without (Gluppe, Engh & Kari 2021; Hills, Graham & McLean 2018; Liaw et al. 2011). According to Beamish et al. (2019), functional impairments associated with the diastasis recti may result from a mechanical deficit related to the stiffness of the linea alba rather than to its increased width. Qualitative structural changes resulting from pregnancy could reduce the linea alba stiffness and negatively affect its function in the management of intra-abdominal pressure, the transfer of force across the midline of the abdomen and the stability of the trunk (Axer et al. 2001; Hodges, Cresswell & Thorstensson 1999; Rath et al. 1996). As a result, postpartum women with an IRD below the threshold value could present a deficiency in abdominal function and may be diagnosed with diastasis recti. Protrusion of the abdomen during contraction of the rectus abdominis muscle has been used to confirm the diastasis recti in postpartum women with a normal IRD (Gluppe et al. 2020; Mahalakshmi et al. 2016). The third aim of our study was to assess the relationships between IRD, linea alba stiffness and abdominal muscle strength and endurance in postpartum women in order to propose recommendations for the diagnosis of diastasis recti and to identify women who would need abdominal rehabilitation.

Thus, in our study we (1) describe the IRD values in three samples of the Beninese population (men and nulliparous and postpartum women); (2) define the IRD threshold values measured at rest using ultrasound and in head lift position using calipers; (3) assess the relationships between IRD, linea alba stiffness, strength and endurance of abdominal muscles; and (4) propose recommendations for diagnosing diastasis recti and for the management of abdominal rehabilitation.

## Method

Our observational cross-sectional study was conducted between March and August 2020, and was carried out at the South of Benin University Hospital called Centre National Hospitalier Universitaire (CNHU) at the Clinique Universitaire de Médecine Physique et Réadaptation (CUMPR). Three categories of participants were enrolled: men and nulliparous and postpartum women. Healthy postpartum women who had given birth more than 6 weeks and less than 6 months previously were recruited while waiting for a consultation in the Physical Medicine and Rehabilitation Department of the University Clinic. Healthy men and nulliparous women were recruited from clinic attendees and from students of the Health Sciences Faculty through an informative note to class delegates.

To be included in our study, participants had to be between 18 and 40 years old. Participants who had undergone abdominal surgery, except caesarean section, were excluded.

### Data collection

#### Interrecti distance measurement

The IRD was measured using ultrasound and calipers. Two physiotherapists – a senior and a junior –trained in measurement techniques carried out the measurements: the junior measured the IRD using the calipers and the senior using ultrasound. The participants were in supine position, with bent knees and arms beside the body. A mark was made at 5 cm above the upper edge of the umbilicus and both measurements were made at this level. The same mark made by the first physiotherapist for the calipers measure was used by the second for the ultrasound measure (Chiarello & McAuley 2013).

#### With calipers

This tool is valid and reliable to measure the IRD (Benjamin et al. 2020; Boxer & Jones 1997; Chiarello & McAuley 2013; Van de Water & Benjamin 2016). The intra-class correlation coefficient (95% confidence interval) for the IRD measurement above the umbilicus is 0.98 (0.95–0.99) in Chiarello and McAuley (2013).

**FIGURE 1:**
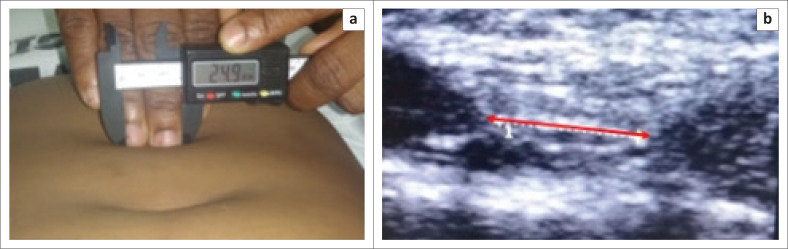
Measurement of interrecti distance: (a) caliper measurement and (b) ultrasound measurement.

The participants lifted their head slightly to contract the rectus abdominis muscle: head lift position (Gluppe et al. 2020). The examiner felt the gap between the right and left rectus abdominis muscles by pushing down gently on the abdomen with her fingers. The IRD was measured by positioning the caliper against the examiner’s finger (Figure 1a).

#### With ultrasound

Ultrasound is also valid and reliable for measuring the IRD (Chiarello & McAuley 2013; Rejano-Campo & Pizzoferrato 2021; Van de Water & Benjamin 2016). The intra-class correlation coefficient (95% confidence interval) for the IRD measurement above the umbilicus is 0.98 (0.92–0.99) (Chiarello & McAuley 2013).

For our study, we used an ultrasound device Chison Eco, 5 MHz –11 MHz wideband Linear Probe. The examiner applied gel to the abdomen and placed the probe transversely over the skin mark without any pressure (Chiarello & McAuley 2013; Rejano-Campo & Pizzoferrato 2021). The image was then captured and the IRD measured, on the screen with the ultrasound calipers, as the space between the two sheaths of the rectus abdominis muscle according to Keshwani, Mathur and McLean (2018) (Figure 1b).

#### Linea alba stiffness measurement

The senior physiotherapist assessed the linea alba stiffness by palpation with the index and the middle fingers. She placed her fingertips on the linea alba perpendicular to the sheath of the rectus abdominis muscle, and then asked the participants to lift their head slightly. The linea alba was considered stiff when a tension was perceived under the fingers, and slack when a gap was perceived between the muscle sheaths.

This measurement has been a daily routine for this physiotherapist for 15 years and was evaluated by a test–retest with 16 postpartum women before the start of our study. The second assessment was made 1 week after the first assessment. The Spearman correlation of the results between the two tests was 1 (*p* < 0.001).

#### Abdominal muscle strength

This variable was measured using manual muscle testing according to Hislop, Avers and Brown (2015): the participants were in supine position with the knees bent and they tried to lift their head and shoulders without moving the pelvis and feet. The testing is rated from 0 to 5 depending on the final position:

0: does not lift at all

1: only lifts the head

2: lifts the head and the upper part of the shoulders with the arms extended towards the knees

3: lifts the head and shoulders up to the point of the scapula with the arms extended towards the knees

4: lifts the head and shoulders to the point of the scapula with the arms crossed on the chest

5: lifts the head and shoulders to the point of the scapula with the hands at the back of the neck.

#### Abdominal muscle endurance

Strength was evaluated using position 2 or 3 of the manual testing described above. The participants maintained the position as long as possible. The measurement was expressed in seconds (Fransoo, Dassain & Mattucci 2009; Ito et al. 1996).

#### Other assessments

- The abdominal skinfold was measured at 2 cm at the right of the umbilicus (Vispute et al. 2011). The measurement was taken using ultrasound as the distance between the superficial part of the skin and the rectus abdominis muscle.
- Abdominal circumference was measured at the umbilicus using a tape measure (Nartea, Mitoiu & Nica 2019).
- Weight and height were measured using a telescopic height scale; these measurements were used to determine the BMI.
- General information about the participants (e.g. age, gender, parity, vaginal or caesarean delivery, and time elapsed since the last childbirth) was collected using a short questionnaire.

### Data analysis

All statistical analyses were performed using SPSS 27 software. The data were not normally distributed. We have therefore presented the results as median with interquartile range (IQR) and carried out non-parametric tests: Mann–Whitney test to compare two independent groups (Tallarida & Murray 1987) and Kruskal–Wallis test to compare three independent groups (Kruskal & Wallis 1952). The level of significance was set at *p* < 0.05 for all tests.

To address the first aim of our study, the IRD for each group was described using the median (IQR). A Kruskal–Wallis test with Bonferroni correction between pairwise comparisons was used to compare the IRD between men and nulliparous and postpartum women.

Regarding the second aim, the IRD threshold was determined:

- For men and nulliparous women as the P90 of the IRD values (Beer et al. 2009; Qu et al. 2021).
- For postpartum women as the P80 of the IRD values measured in primiparous postpartum women (Mota et al. 2018).

Regarding the third aim that specifically concerned the sample of postpartum women:

- The receiver operating characteristics (ROC) curve was used as a representation of specificity and sensitivity to define an IRD threshold value based on the linea alba stiffness. Each point in the ROC curve defines an IRD threshold with the corresponding sensitivity and specificity. We chose the threshold that simultaneously offered the maximum sensitivity and specificity.
- Postpartum women with an IRD above and below the P80 threshold value were compared based on the linea alba stiffness, abdominal strength and abdominal endurance. Similarly, women with a stiff linea alba were compared to those with a slack linea alba based on IRD values, abdominal strength and abdominal endurance. The Mann–Whitney test was used for these comparisons.

### Ethical considerations

Ethical approval to conduct the study was obtained from the Ethics Committee of CUMPR (N°01- 2020/MS/CNHU-HKM/CER/CUMPR). Experimental procedures were performed in accordance with the Declaration of Helsinki as revised in 2013. All participants completed a written consent form prior to participating in our study.

## Results

### Sample characteristics

A total of 391 participants were enrolled in our study: 80 men, 158 nulliparous women and 153 postpartum women (vaginal delivery and caesarean, parity 1–6). The characteristics of the participants in the three samples of the Beninese population are summarised in Table 1.

**TABLE 1:** Characteristics of participants in the three samples of the Beninese population (*n* = 391).

Variable	Nulliparous women (*n* = 158)		Men (*n* = 80)		Postpartum women (*n* = 153)	
	Mean	IQR	Mean	IQR	Mean	IQR
Age (Years)	22	4	23	4	31	10
Weight (kg)	60	11	64	8	68	14
Height (cm)	161	6	173	7	163	7
Bill (kg/m^2^)	23	5	21	30	26	6
Ombilical circumference (cm)	74	12	72	10	85	17
Abdominal skinfold (mm)	11	8	6	6	14	11
Abdominal strength (0–5)	5	1	5	0	3	2
Abdominal endurance (sec)†	116	98	158	53	52	53
IRD with ultrasound (mm)	12	5	14	6	21	12
IRD with caliper (mm)	11	8	13	10	18	12

BMI, body mass index; IRD, interrecti distance; IQR, interquartile range.

† , *n* = 142 (postpartum women); 11 missing data.

### Interrecti distance median values in the three samples of the Beninese population

The IRD median (IQR) values in men, nulliparous women and postpartum women were, respectively, 14 mm (6), 12 mm (5) and 21 mm (12) for ultrasound measurement at rest, and 13 mm (10), 11 mm (8) and 18 mm (12) for calipers measurement in the head lift position (Table 1). The IRD was significantly greater (*H* = 170.7; degrees of freedom (*df)* = 2; *p* < 0.001) in postpartum women than in men and nulliparous women. There were no statistically significant differences between men and nulliparous women at rest (ultrasound measurement) or in the head lift position (calipers measurement).

### Interrecti distance threshold values in the three samples of the Beninese population

In men, the P90 of the IRD values was 20 mm at rest (ultrasound measurement) and 17 mm in the head lift position (calipers measurement).

In nulliparous women, the P90 of the IRD values was 18 mm at rest (ultrasound measurement) and 15 mm in the head lift position (calipers measurement).

In postpartum women, the P80 of the IRD values measured in the 20 primiparous women who had given birth 3–6 months earlier was 23 mm at rest (ultrasound measurement) and 18 mm in the head lift position (calipers measurement). Applying this IRD threshold value to all postpartum women, we observed a diastasis recti prevalence of 46%.

### Relationships between interrecti distance, linea alba stiffness, abdominal muscle strength and endurance in the sample of postpartum women

**FIGURE 2:**
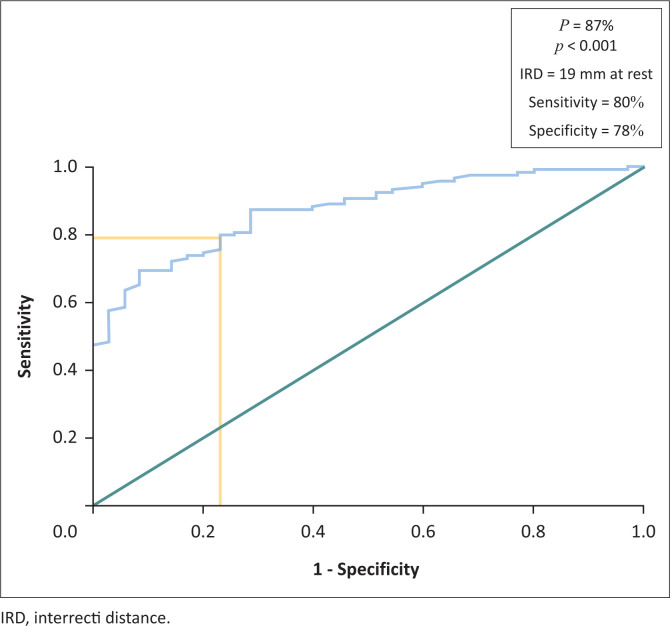
Receiver operating characteristics curve for determination of normal interrecti distance according to the linea alba stiffness (*n* = 153).

Based on the evaluation of the linea alba stiff or slack, an IRD threshold value was defined using the ROC curve (Figure 2). We chose the threshold that simultaneously offered 80% sensitivity and 78% specificity with a power of 87%. This threshold corresponds to 19 mm with ultrasound and 15 mm with calipers. This means that 80% of the postpartum women with a slack linea alba had an IRD greater than 19 mm at rest and 15 mm in the head lift position; and 78% of the postpartum women with an IRD greater than 19 mm at rest and 15 mm in the head lift position had a slack linea alba.

Table 2 summarises the characteristics of postpartum women whose IRD was, respectively, lower or higher than the P80 threshold value (Table 2, left columns), and whose linea alba was, respectively, slack or stiff (Table 2, right columns). Abdominal strength was significantly lower (*p* = 0.02) in women with an IRD above rather than below the threshold value. Abdominal strength (*p* = 0.001) and abdominal endurance (*p* = 0.001) were significantly lower and the IRD (*p* < 0.001) was significantly larger in women with a slack rather than a stiff linea alba. While most of the women (69 of 71) with an IRD above the threshold value had a slack linea alba, only 40% (33 of 82) of the women with an IRD below the threshold value had a stiff linea alba. Moreover, among the women with an IRD below the P80 threshold value, those with a slack linea alba had significantly lower abdominal muscles strength and endurance compared to those with a stiff linea alba. Their abdominal strength was, respectively, 3 (1) versus 4 (1) and their endurance was, respectively, 43 (36) s versus 70 (50) s.

**TABLE 2:** Relationships between IRD, linea alba stiffness, abdominal muscle strength and endurance in the sample of postpartum women (*n* = 153).

Variable	IRD				Linea alba			
	≤ P80		> P80		Stiff		Slack	
	Median	IQR	Median	IQR	Median	IQR	Median	IQR
Abdominal strength (0–5)	3	2	2	2*	4	1	2	2*
Abdominal endurance (sec)†	61	57	56	28	72	56	54	34*
IRD with ultrasound (mm)	-	-	-	-	16	5	26	11*
IRD with caliper (mm)	-	-	-	-	12	5	21	11*
Linea alba								
Stiff (*n*)	33	-	2	-	-	-	-	-
Slack (*n*)	49	-	69	-	-	-	-	-

**Total (*n*)**	**-**	**82**	**-**	**71**	**-**	**35**	**-**	**118**

BMI, body mass index; IRD, interrecti distance; IQR, interquartile range.

P80 = 23 mm at rest with ultrasound or 18 mm in head lift with caliper.

* , Significant difference;

† , *n* = 142 (11 missing data).

## Discussion

The IRD values were measured 5 cm above the umbilicus. Postpartum women had greater IRD values than men and nulliparous women. In men and nulliparous women, the P90 of the IRD values was, respectively, 20 mm and 18 mm measured at rest using ultrasound, and 17 mm and 15 mm measured in the head lift position using the calipers. In primiparous women, the P80 of the IRD values was 23 mm at rest and 18 mm in the head lift position. With this threshold (P80), the diastasis recti prevalence among all postpartum women was 46%. Using the linea alba stiffness, a new threshold value of 19 mm at rest and 15 mm in the head lift position was defined in postpartum women. A slack linea alba was associated with lower abdominal muscle function, even in women with a normal IRD value.

The median IRD values measured at rest using ultrasound were 12 ± 5 mm in men, 14 ± 6 mm in nulliparous women and 21 ± 12 mm in postpartum women. In men, Chiarello et al. (2016) measured 16.2 ± 10.4 mm close to our median (IQR) value, while Lee and Hodges (2016) and Qu et al. (2021) measured much lower values, respectively, 8.0 ± 3.8 mm and 6.2 ± 5.6 mm. In nulliparous women, Beer et al. (2009) measured 13 ± 7 mm similar to our median value, while Chiarello et al. (2016) and Lee and Hodges (2016) measured much lower values, respectively, 7.5 ± 4.3 mm and 7.7 ± 3.4 mm. In postpartum women, Mota et al. (2018) measured 23 ± 9.3 mm at 6 weeks and 18.7 ± 8.4 mm at 6 months postpartum, Qu et al. (2021) measured 23.6 ± 8.6 mm and Chiarello et al. (2016) measured 20.3 ± 10.5 mm. Our median values were close to the mean values measured in these three studies but much lower than the 42.2 ± 9.7 mm measured by Ojukwu et al. (2021). The large disparity between the IRD values measured in these studies can be partially explained by the level of the measurement along the linea alba. For instance, the very high values observed by Ojukwu et al. (2021) were measured at the level of the umbilicus, where the IRD is known to be the largest (Rath et al. 1996). Nevertheless, this factor cannot explain most of the observed differences, and it would be worthwhile to investigate other potential predictors of IRD values in different populations.

We compared the IRD values among the three samples of participants. We did not observe a significant difference between men and nulliparous women, consistent with the observations of Lee and Hodges (2016). In contrast, Chiarello et al. (2016) observed a significantly larger IRD value in men compared to nulliparous women. In their study, the men were older and had a greater BMI than the nulliparous women, which suggests that age and BMI could influence IRD values.

Significantly larger IRD values were reported in postpartum women than in nulliparous women. The same observation was made by Chiarello and McAuley (2013) and Lee and Hodges (2016). The high IRD values measured at the end of pregnancy progressively decrease during the first few months after childbirth but never return to pre-pregnancy values (Coldron et al. 2008; Liaw et al. 2011; Mota et al. 2018), which suggests that different thresholds should be used to define IRD for nulliparous and postpartum women.

The IRD threshold values that we defined using the calipers measurement can serve as a reference for physiotherapists in Benin to diagnose the diastasis recti in nulliparous and postpartum women and in men. To date the issue of the diastasis recti in men has been sparsely studied, but it could deserve more attention in the future (Nienhuijs et al. 2021).

The IRD threshold values measured at rest with ultrasound can be compared to already published values in other population samples. In nulliparous women, the IRD was normal if it was less than 18 mm. In two similar populations of young (under 30 years old) nulliparous women with a BMI under 25, the P90 threshold value was 22 mm (Beer et al. 2009) and 14 mm (Qu et al. 2021). In these two studies the measurements were taken 3 cm above the umbilicus rather than 5 cm in our study. Larger IRD values are usually observed closer to the umbilicus (Axer et al. 2001; Beer et al. 2009; Lee & Hodges 2016; Mota et al. 2015), which may explain the difference between the values measured by Beer et al. (2009) and our results, but cannot explain the smaller values measured by Qu et al. (2021). Possible ethnicity-related differences should be investigated as one study was conducted in Europe (Beer et al. 2020), one other in China (Qu et al. 2021) and ours in Africa. These differences could be related to the role played by collagen fibres on the appearance of diastasis recti and hernias (Blotta et al. 2018; Mosanya et al. 2020). Different proportions of collagen fibres have been reported in the anterior rectus sheath of different ethnic populations, namely, an African Nigerian population (Mosanya et al. 2020), South-American Brazilian population (Casanova, Trindade & Trindade 2009; Gonçalves, De Moraes E Silva & Lopes Filho 2014) and a European Portuguese population (Blotta et al. 2018). To move forward on that issue, a study should be designed to compare different ethnic populations regarding histological characteristics of the abdominal wall in postpartum women with and without diastasis recti.

In postpartum women, the IRD was normal when less than 23 mm, a value close to the 24 mm measured in similar conditions in a European population sample by Mota et al. (2018). A subtle difference can nevertheless be noticed related to the shorter time elapsed since delivery in our study (3–6 months) compared to the study of Mota et al. (2018) (6 months). As the IRD spontaneously decreases during the first 6 months after pregnancy (Liaw et al. 2011; Mota et al. 2018), it could be hypothesised that at 6 months after delivery the P80 IRD value would have been smaller in our study than the value measured by Mota et al. (2018).

Regarding the prevalence of diastasis recti in postpartum women, our results collected on women of African ethnicity can be compared to those collected on women of European ethnicity in the study by Sperstad et al. (2016). In both studies, the IRD was measured about 5 cm above the umbilicus during rectus abdominis muscle contraction and the reported prevalence was about 45%. However, the 18 mm threshold value that we used to diagnose diastasis recti was much lower than the 2-finger breadth threshold used by Sperstad et al. (2016) as a finger corresponds to 15 mm according to Turan et al. (2011) and Rett et al. (2009). Applied to our sample of postpartum women, this 2-finger breadth threshold would have resulted in a much lower prevalence. Moreover, our sample had several differences with the sample examined in Sperstad et al. (2016); in our sample, the average parity was higher (we examined both primiparous and multiltiparous women, while Sperstad et al. (2016) studied only primiparous women), and the average time since delivery in our study was shorter (our sample ranged from 6 weeks to 6 months, while Sperstad et al. (2016) examined only the 6 months timepoint). As a result, a higher prevalence would have been expected in our population sample compared to that of Sperstad et al. (2016).

In 2007, Spitznagle, Leong and Van Dillen observed the prevalence of diastasis recti in a urogynaecological population consisting of 539 patients of different ethnic groups. The prevalence was significantly lower among African-American women (33%) than among white women (58%). This observation together with our comparison with the study of Sperstad et al. (2016) (see the paragraph above) supports the hypothesis that the prevalence of diastasis recti could be ethnicity related. However, the small sample of primiparous women in our study and the small percentage (14%) of African-American women in the study conducted in North America (Spitznagle et al. 2007) do not allow us to confirm this hypothesis right now. It should be investigated in future studies with a larger number of participants.

Postpartum women diagnosed with diastasis recti (IRD above the P80 threshold) had less strength than those without, as previously observed by several researchers (Gluppe et al. 2021; Gunnarsson et al. 2015; Hills et al. 2018). We used palpation to measure the linea alba stiffness in a binary ‘stiff’ versus ‘slack’ evaluation, and we observed that a slack linea alba was associated with less abdominal strength and endurance, even in women without diastasis recti. Among the postpartum women with a normal IRD, 60% had a slack linea alba. This justifies considering the linea alba stiffness for the diagnosis of diastasis recti and for the decision to offer abdominal rehabilitation. Based on linea alba stiffness, a new IRD threshold value, which was lower than the P80 threshold, was defined through the ROC curve with both a high sensitivity and a high specificity.

We propose to consider this new threshold in the clinical evaluation of postpartum women and to pay close attention to women with an IRD below the P80 threshold but above this new stiffness-based threshold. Linea alba stiffness and abdominal strength and endurance should be evaluated in these women to identify their need for abdominal rehabilitation. Some of them with very poor abdominal function could even be diagnosed with diastasis recti. Considering the IRD increase with parity (Gitta et al. 2017) and the greater IRD reduction observed after a rehabilitation programme in women with a parity of 2 compared to those with a parity of 4 or 5 (Igwe et al. 2020), abdominal rehabilitation should be recommended after each delivery, especially in the Beninese population whose average birth rate per woman is 5 (Leridon 2015).

To diagnose diastasis recti, parameters such as parity, linea alba stiffness, abdominal muscle strength and endurance must be considered besides IRD. In some cases, women may need abdominal rehabilitation even if their IRD is below the diastasis recti threshold value. Hereafter we propose a simple approach allowing a physiotherapist to diagnose diastasis recti and to identify the need for abdominal care through a two-step analysis. Firstly, the decision to treat is based on the comparison of the woman IRD value with a threshold value specific to her obstetrical status, parous or nulliparous. Secondly, for parous women with specific IRD values as defined in the first step, the evaluation of their abdominal function contributes to treatment decisions. This reasoning is proposed as a clinical approach, IRD values will be those measured with calipers.

First step: parous or nulliparous?

- If nulliparous, there is no diastasis recti if the IRD is less than 15 mm (P90 of nulliparous women). When the IRD is greater than 15 mm, this woman may be at risk of developing the diastasis recti in future pregnancies. Antenatal IRD reduction could be considered.
- If parous:
  ■ There is no diastasis recti and no need for abdominal rehabilitation if the IRD is less than 15 mm (ROC curve).
  ■ When the IRD is greater than 18 mm (P80 of primiparous postpartum women), the woman is diagnosed with diastasis recti and should benefit from abdominal rehabilitation.
  ■ When the IRD is between 15 mm (ROC curve) and 18 mm (P80), the linea alba stiffness, abdominal muscle strength and endurance should be evaluated (see the second step hereafter).

Second step: how well do the woman’s abdominal muscles function?

**FIGURE 3:**
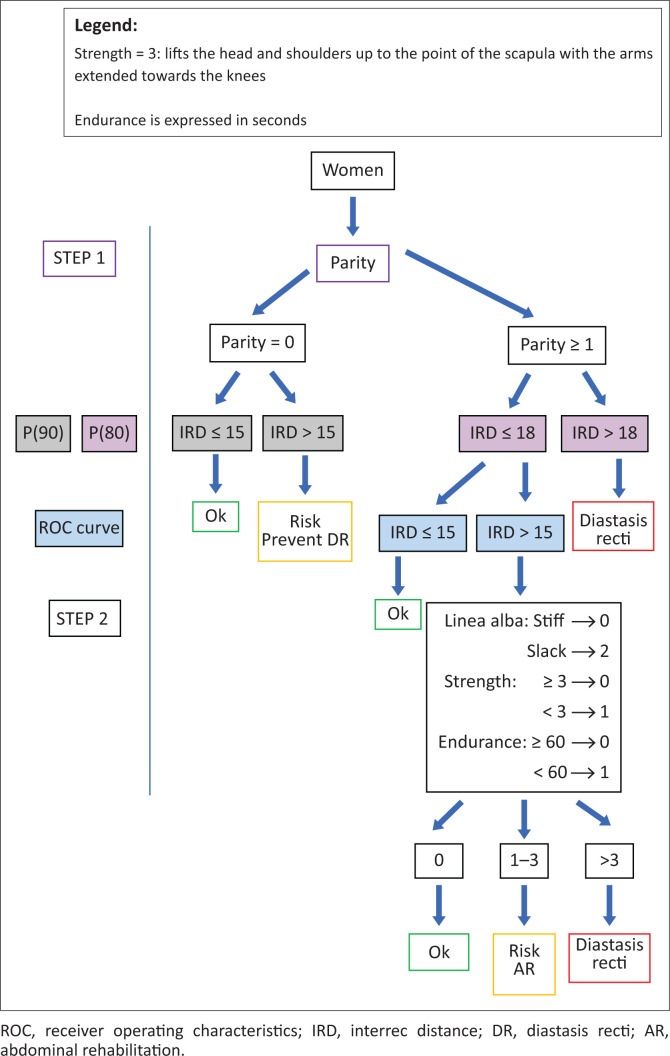
Decision tree for diastasis recti diagnosis and decision for abdominal rehabilitation treatment considering the interrecti distance measured at 5 cm above the umbilicus in a head lift position.

- If her linea alba is stiff, her abdominal muscle strength reaches at least 3 (see the legend in Figure 3) and her abdominal muscle endurance reaches at least 60 s, the woman does not require abdominal treatment.
- If her linea alba is slack or her abdominal muscle strength or endurance is low, the woman may be at risk of developing the diastasis recti in future pregnancies and could benefit from treatment.
- If her linea alba is slack, her abdominal muscle strength is under 3 and her abdominal muscle endurance does not reach 60 s, the woman has diastasis recti.

### Strengths and limitations of our study

Our study provides IRD values for three samples of the Beninese population. Interrecti distance threshold values were defined using calipers, a tool commonly used in clinical practice. A new IRD threshold value was proposed based on the stiffness of the linea alba. A decision tree based on functional parameters was proposed to help physiotherapists in the diagnosis of the diastasis recti and in the decision for abdominal rehabilitation.

The P80 threshold value used for diagnosing diastasis recti in the postpartum women was defined based on the IRD values of the 20 primiparous women. Other studies with larger numbers of primiparous women should confirm values measured in our study. All participants lived in or around Cotonou and had a moderate to high level of education, which is usually related to a higher age of first pregnancy and a lower number of children. This does not allow generalisation of the findings to the entire population of the country.

## Conclusion

Interrecti distance median and threshold values specific to Beninese men and nulliparous and postpartum women were defined using ultrasound and calipers. Based on a threshold value of 23 mm at rest and 18 mm in a head lift position, a diastasis recti prevalence of 46% was observed in postpartum women. Differences in IRD values or in prevalence with other studies can be related to differences in the methodology of measurement or in population samples. Possible ethnicity-related differences cannot be excluded and should be investigated further.

In postpartum women, a slack linea alba was associated with lower abdominal strength and endurance even in women with a normal IRD value. Based on this observation, a decision-tree approach was built that combines the measurement of IRD, linea alba stiffness and abdominal strength and endurance to help physiotherapists in evaluating a woman’s need for abdominal rehabilitation. Future randomised controlled trials should investigate the effects of specific rehabilitation protocols designed to reduce IRD, restore linea alba stiffness and increase abdominal muscle strength and endurance.

## References

[CIT0001] Akram, J. & Matzen, S.H., 2014, ‘Rectus abdominis diastasis’, *Journal of Plastic Surgery and Hand Surgery* 48(3), 163–169. 10.3109/2000656X.2013.859145 24256310

[CIT0002] Alamer, A., Kahsay, G. & Ravichandran, H., 2019, ‘Prevalence of diastasis recti and associated factors among women attending antenatal and postnatal careatmekelle city health facilities, Tigray, Ethiopia’, *Age* 20, 37.

[CIT0003] Axer, H., Keyserlingk, D.G.V. & Prescher, A., 2001, ‘Collagen fibers in linea alba and rectus sheaths: I. General scheme and morphological aspects’, *Journal of Surgical Research* 96(1), 127–134. 10.1006/jsre.2000.6070 11181006

[CIT0004] Beamish, N., Green, N., Nieuwold, E. & McLean, L., 2019, ‘Differences in linea alba stiffness and linea alba distortion between women with and without diastasis recti abdominis: The impact of measurement site and task’, *Journal of Orthopaedic & Sports Physical Therapy* 49(9), 656–665. 10.2519/jospt.2019.8543 30913968

[CIT0005] Beer, G.M., Schuster, A., Seifert, B., Manestar, M., Mihic-Probst, D. & Weber, S.A., 2009, ‘The normal width of the linea alba in nulliparous women’, *Clinical Anatomy* 22(6), 706–711. 10.1002/ca.20836 19637295

[CIT0006] Benjamin, D.R., Frawley, H.C., Shields, N., Georgiou, C. & Taylor, N.F., 2020, ‘Establishing measurement properties in the assessment of inter-recti distance of the abdominal muscles in a postnatal women’, *Musculoskeletal Science and Practice* 49, 102202. 10.1016/j.msksp.2020.102202 32861363

[CIT0007] Blotta, R.M., Costa, S.D.S., Trindade, E.N., Meurer, L. & Maciel-Trindade, M.R., 2018, ‘Collagen I and III in women with diastasis recti’, *Clinics* 73, e319. 10.6061/clinics/2018/e319 29898006 PMC5971415

[CIT0008] Boissonnault, J.S. & Blaschak, M.J., 1988, ‘Incidence of diastasis recti abdominis during the childbearing year’, *Physical Therapy* 68(7), 1082–1086. 10.1093/ptj/68.7.1082 2968609

[CIT0009] Boxer, S. & Jones, S., 1997, ‘Intra-rater reliability of rectus abdominis diastasis measurement using dial calipers’, *Australian Journal of Physiotherapy* 43(2), 109–114. 10.1016/S0004-9514(14)60405-0 11676678

[CIT0010] Casanova, A.B., Trindade, E.N. & Trindade, M.R.M., 2009, ‘Collagen in the transversalis fascia of patients with indirect inguinal hernia: A case-control study’, *The American Journal of Surgery* 198(1), 1–5. 10.1016/j.amjsurg.2008.07.021 19095216

[CIT0011] Chiarello, C.M. & McAuley, J.A., 2013, ‘Concurrent validity of calipers and ultrasound imaging to measure interrecti distance’, *Journal of Orthopaedic & Sports Physical Therapy* 43(7), 495–503. 10.2519/jospt.2013.4449 23633625

[CIT0012] Chiarello, C.M., McAuley, J.A. & Hartigan, E.H., 2016, ‘Immediate effect of active abdominal contraction on inter-recti distance’, *Journal of Orthopaedic & Sports Physical Therapy* 46(3), 177–183. 10.2519/jospt.2016.6102 26813756

[CIT0013] Coldron, Y., Stokes, M.J., Newham, D.J. & Cook, K., 2008, ‘Postpartum characteristics of rectus abdominis on ultrasound imaging’, *Manual Therapy* 13(2), 112–121. 10.1016/j.math.2006.10.001 17208034

[CIT0014] Fransoo, P., Dassain, C. & Mattucci, P., 2009, ‘Mise en pratique du test de Shirado: Implementation of the Shirado test’, *Kinésithérapie, la Revue* 9(87), 39–42. 10.1016/S1779-0123(09)70777-6

[CIT0015] Gilleard, W.L. & Brown, J.M.M., 1996, ‘Structure and function of the abdominal muscles in primigravid subjects during pregnancy and the immediate postbirth period’, *Physical Therapy* 76(7), 750–762. 10.1093/ptj/76.7.750 8677279

[CIT0016] Gitta, S., Magyar, Z., Tardi, P., Fuge, I., Jaromi, M., Acs, P. et al., 2017, ‘Prevalence, potential risk factors and sequelae of diastasis recti abdominis’, *Orvosi Hetilap* 158(12), 454–460. 10.1556/650.2017.30703 28328249

[CIT0017] Gluppe, S.B., Engh, M.E. & Bø, K., 2020, ‘Immediate effect of abdominal and pelvic floor muscle exercises on interrecti distance in women with diastasis recti abdominis who were parous’, *Physical Therapy* 100(8), 1372–1383. 10.1093/ptj/pzaa070 32302393

[CIT0018] Gluppe, S., Engh, M.E. & Kari, B., 2021, ‘Women with diastasis recti abdominis might have weaker abdominal muscles and more abdominal pain, but no higher prevalence of pelvic floor disorders, low back and pelvic girdle pain than women without diastasis recti abdominis’, *Physiotherapy* 111, 57–65. 10.1016/j.physio.2021.01.008 33691943

[CIT0019] Gonçalves, R.D.O., De Moraes E Silva, E. & Lopes Filho, G.D.J., 2014, ‘Immunohistochemical evaluation of fibrillar components of the extracellular matrix of transversalis fascia and anterior abdominal rectus sheath in men with inguinal hernia’, *Revista do Colégio Brasileiro de Cirurgiões* 41(1), 23–29. 10.1590/S0100-69912014000100006 24770770

[CIT0020] Gunnarsson, U., Stark, B., Dahlstrand, U. & Strigard, K., 2015, ‘Correlation between abdominal rectus diastasis width and abdominal muscle strength’, *Digestive Surgery* 32(2), 112–116. 10.1159/000371859 25766128

[CIT0021] Hills, N.F., Graham, R.B. & McLean, L., 2018, ‘Comparison of trunk muscle function between women with and without diastasis recti abdominis at 1 year postpartum’, *Physical Therapy* 98(10), 891–901. 10.1093/ptj/pzy083 30011041

[CIT0022] Hislop, H., Avers, D. & Brown, M., 2015, *Le bilan musculaire de Daniels et Worthingham: Technique de testing manuel*, Elsevier Masson SAS, France, Issy-les-Moulineaux

[CIT0023] Hodges, P., Cresswell, A. & Thorstensson, A., 1999, ‘Preparatory trunk motion accompanies rapid upper limb movement’, *Experimental Brain Research* 124, 69–79. 10.1007/s002210050601 9928791

[CIT0024] Igwe, S.E. & Okoye, G.C., 2020, ‘Abdominal exercises enhance closure of diastasis recti abdominis condition and improves quality of life among women who have undergone multiple pregnancies’, *Academic Journal of Current Research* 7, 36–45.

[CIT0025] Igwe, S.E., Okoye, G.C. & Chukwu, S.C., 2020, ‘The impact of parity and age in exercice mediated resolution of diastasis recti abdominis among postpartum women: A survey of women in Abakaliki Eastern Nigeria’, *Academic Journal of Current Research* 7(6), 87–98.

[CIT0026] Ito, T., Shirado, O., Suzuki, H., Takahashi, M., Kaneda, K. & Strax, T.E., 1996, ‘Lumbar trunk muscle endurance testing: An inexpensive alternative to a machine for evaluation’, *Archives of Physical Medicine and Rehabilitation* 77, 75–79. 10.1016/S0003-9993(96)90224-5 8554479

[CIT0027] Keshwani, N., Mathur, S. & McLean, L., 2018, ‘Relationship between interrectus distance and symptom severity in women with diastasis recti abdominis in the early postpartum period’, *Physical Therapy* 98(3), 182–190. 10.1093/ptj/pzx117 29228344

[CIT0028] Kruskal, W.H. & Wallis, W.A., 1952, ‘Use of ranks in one-criterion variance analysis’, *Journal of the American Statistical Association* 47(260), 583–621. 10.1080/01621459.1952.10483441

[CIT0029] Lee, D. & Hodges, P.W., 2016, ‘Behavior of the linea alba during a curl-up task in diastasis rectus abdominis: An observational study’, *Journal of Orthopaedic & Sports Physical Therapy* 46(7), 580–589. 10.2519/jospt.2016.6536 27363572

[CIT0030] Leridon, H., 2015, ‘Afrique subsaharienne: Une transition démographique explosive’, *Futuribles* 407, 5–21.

[CIT0031] Liaw, L.J., Hsu, M.J., Liao, C.F., Liu, M.F. & Hsu, A.T., 2011, ‘The relationships between inter-recti distance measured by ultrasound imaging and abdominal muscle function in postpartum women: A 6-month follow-up study’, *Journal of Orthopaedic & Sports Physical Therapy* 41(6), 435–443. 10.2519/jospt.2011.3507 21289454

[CIT0032] Mahalakshmi, V., Sumathi, G., Chitra, T. & Ramamoorthy, V., 2016, ‘Effect of exercise on diastasis recti abdominis among the primiparous women: A quasi-experimental study’, *International Journal of Reproduction, Contraception, Obstetrics and Gynecology* 5(12), 4441–4446. 10.18203/2320-1770.ijrcog20164360

[CIT0033] Mosanya, A., Olasehinde, O., Odujoko, O., Etonyeaku, A., Adumah, C. & Agbakwuru, E., 2020, ‘Comparative study of collagen and elastin content of abdominal wall fascia in inguinal hernia and non-hernia patients in an African population’, *Hernia* 24, 1337–1344. 10.1007/s10029-020-02238-y 32488528

[CIT0034] Mota, P., Pascoal, A.G., Carita, A.I. & Bo, K., 2015, ‘The immediate effects on inter-rectus distance of abdominal crunch and drawing-in exercises during pregnancy and the postpartum period’, *Journal of Orthopaedic & Sports Physical Therapy* 45(10), 781–788. 10.2519/jospt.2015.5459 26304639

[CIT0035] Mota, P., Pascoal, A.G., Carita, A.I. & Bo, K., 2018, ‘Normal width of the inter-recti distance in pregnant and postpartum primiparous women’, *Musculoskeletal Science and Practice* 35, 34–37. 10.1016/j.msksp.2018.02.004 29494833

[CIT0036] Nartea, R., Mitoiu, B.I. & Nica, A.S., 2019, ‘Correlation between pregnancy related weight gain, postpartum weight loss and obesity: A prospective study’, *Journal of Medicine and Life* 12, 178. 31406521 10.25122/jml-2019-0015 PMC6685304

[CIT0037] Nienhuijs, S., Berkvens, E., De Vries Reilingh, T., Mommers, E., Bouvy, N. & Wegdam, J., 2021, ‘The male rectus diastasis: A different concept?’, *Hernia* 25, 951–956. 10.1007/s10029-021-02467-9 34297251

[CIT0038] Ojukwu, C., Ezeigwe, A., Ezeigwe, C., Onuchukwu, C. & Agwagu, I., 2021, ‘Correlates of inter-rectus distance in Nigerian parous women’, *International Journal of Medicine and Health Development* 26(2), 123–127. 10.4103/ijmh.IJMH_52_20

[CIT0039] Pascoal, A.G., Dionisio, S., Cordeiro, F. & Mota, P., 2014, ‘Inter-rectus distance in postpartum women can be reduced by isometric contraction of the abdominal muscles: A preliminary case-control study’, *Physiotherapy* 100(4), 344–348. 10.1016/j.physio.2013.11.006 24559692

[CIT0040] Qu, E., Wu, J., Zhang, M., Wu, L., Zhang, T., Xu, J. et al., 2021, ‘The ultrasound diagnostic criteria for diastasis recti and its correlation with pelvic floor dysfunction in early postpartum women’, *Quantitative Imaging in Medicine and Surgery* 11(2), 706–713. 10.21037/qims-20-596 33532270 PMC7779936

[CIT0041] Rath, A., Attali, P., Dumas, J., Goldlust, D., Zhang, J. & Chevrel, J., 1996, ‘The abdominal linea alba: An anatomo-radiologic and biomechanical study’, *Surgical and Radiologic Anatomy* 18, 281–288. 10.1007/BF01627606 8983107

[CIT0042] Rejano-Campo, M. & Pizzoferrato, A.-C., 2021, ‘Échographie en rééducation pelvi-périnéale: Quelles applications?’, *Kinésithérapie, la Revue* 21(229), 25–32. 10.1016/j.kine.2020.06.011

[CIT0043] Rett, M., Braga, M., Bernardes, N. & Andrade, S., 2009, ‘Prevalence of diastasis of the rectus abdominis muscles immediately postpartum: Comparison between primiparae and multiparae’, *Brazilian Journal of Physical Therapy* 13(4), 275–280. 10.1590/S1413-35552009005000037

[CIT0044] Sancho, M.F., Pascoal, A.G., Mota, P. & Bo, K., 2015, ‘Abdominal exercises affect inter-rectus distance in postpartum women: A two-dimensional ultrasound study’, *Physiotherapy* 101(3), 286–291. 10.1016/j.physio.2015.04.004 26094117

[CIT0045] Sperstad, J.B., Tennfjord, M.K., Hilde, G., Ellstrom-Engh, M. & Bo, K., 2016, ‘Diastasis recti abdominis during pregnancy and 12 months after childbirth: Prevalence, risk factors and report of lumbopelvic pain’, *British Journal of Sports Medicine* 50(17), 1092–1096. 10.1136/bjsports-2016-096065 27324871 PMC5013086

[CIT0046] Spitznagle, T.M., Leong, F.C. & Van Dillen, L.R., 2007, ‘Prevalence of diastasis recti abdominis in a urogynecological patient population’, *International Urogynecology Journal and Pelvic Floor Dysfunction* 18, 321–328. 10.1007/s00192-006-0143-5 16868659

[CIT0047] Tallarida, R.J. & Murray, R.B., 1987, ‘Mann-whitney test’, in *Manual of pharmacologic calculations*, New York: Springer, p. 149–153.

[CIT0048] Turan, V., Colluoglu, C., Turkuilmaz, E. & Korucuoglu, U., 2011, ‘Prevalence of diastasis recti abdominis in the population of young multiparous adults in Turkey’, *Ginekologia Polska* 82(11), 817–821. 22384613

[CIT0049] Van de Water, A.T. & Benjamin, D.R., 2016, ‘Measurement methods to assess diastasis of the rectus abdominis muscle (DRAM): A systematic review of their measurement properties and meta-analytic reliability generalisation’, *Manual Therapy* 21, 41–53. 10.1016/j.math.2015.09.013 26474542

[CIT0050] Vispute, S.S., Smith, J.D., Lecheminant, J.D. & Hurley, K.S., 2011, ‘The effect of abdominal exercise on abdominal fat’, *The Journal of Strength & Conditioning Research* 25(9), 2559–2564. 10.1519/JSC.0b013e3181fb4a46 21804427

